# PacBio Hi-Fi genome assembly of *Sipha maydis*, a model for the study of multipartite mutualism in insects

**DOI:** 10.1038/s41597-024-03297-x

**Published:** 2024-05-04

**Authors:** François Renoz, Nicolas Parisot, Patrice Baa-Puyoulet, Léo Gerlin, Samir Fakhour, Hubert Charles, Thierry Hance, Federica Calevro

**Affiliations:** 1 Biodiversity Research Centre, Earth and Life Institute, UCLouvain, Louvain-la-Neuve 1348 Belgium; 2 Univ Lyon, INSA Lyon, INRAE, BF2I, UMR203, Villeurbanne, F-69621 France; 3 Institute of Agrobiological Sciences, National Agriculture and Food Research Organization (NARO), Tsukuba, Ibaraki 305-8634 Japan; 4 Univ Lyon, INRAE, INSA Lyon, BF2I, UMR203, Villeurbanne, F-69621 France; 5 Department of Plant Protection, National Institute for Agricultural Research (INRA), Béni-Mellal, 23000 Morocco

**Keywords:** Entomology, Genomic analysis, Biochemical reaction networks, Biochemical networks

## Abstract

Dependence on multiple nutritional endosymbionts has evolved repeatedly in insects feeding on unbalanced diets. However, reference genomes for species hosting multi-symbiotic nutritional systems are lacking, even though they are essential for deciphering the processes governing cooperative life between insects and anatomically integrated symbionts. The cereal aphid *Sipha maydis* is a promising model for addressing these issues, as it has evolved a nutritional dependence on two bacterial endosymbionts that complement each other. In this study, we used PacBio High fidelity (HiFi) long-read sequencing to generate a highly contiguous genome assembly of *S. maydis* with a length of 410 Mb, 3,570 contigs with a contig N50 length of 187 kb, and BUSCO completeness of 95.5%. We identified 117 Mb of repetitive sequences, accounting for 29% of the genome assembly, and predicted 24,453 protein-coding genes, of which 2,541 were predicted enzymes included in an integrated metabolic network with the two aphid-associated endosymbionts. These resources provide valuable genetic and metabolic information for understanding the evolution and functioning of multi-symbiotic systems in insects.

## Background & Summary

Nutritional symbiosis with bacteria has contributed significantly to the evolutionary success of insect taxa that feed on unbalanced diets such as phloem sap, blood or wood^1^. Indeed, in many insect species, the synthesis of nutrients (e.g. amino acids and/or vitamins) that are not present in sufficient amounts in the diet is ensured by an obligate nutritional symbiont, sometimes acquired tens of millions of years ago^2^. These symbionts are generally transmitted faithfully from generation to generation (i.e., by vertical transmission), and compartmentalized in specific host cells called bacteriocytes^3^. These cells mediate the metabolic exchanges between the insect and its bacterial partners, and regulate populations of obligate symbionts according to the insect’s nutritional needs throughout its life cycle^4,5^. However, this intracellular lifestyle causes Muller’s ratchet which, combined with severe population bottlenecks during vertical transmission and the relaxation of purifying selection on genes no longer needed in the context of interdependent association, leads to reductive genome evolution^6^. In some insect lineages, the ancestral nutritional symbiont has undergone such severe genomic erosion that it is no longer able to supply alone the compounds essential to its host’s physiology on its own, and is metabolically complemented by more recently acquired nutritional symbionts^1,7^. Thus, in many insect taxa, nutritional symbiosis does not rely on a single obligate symbiont but on a consortium of nutritional symbionts that evolve together in the same host, either within the same bacteriocytes^8–11^, or in distinct but anatomically connected bacteriocytes^12–16^.

**Fig. 1:**
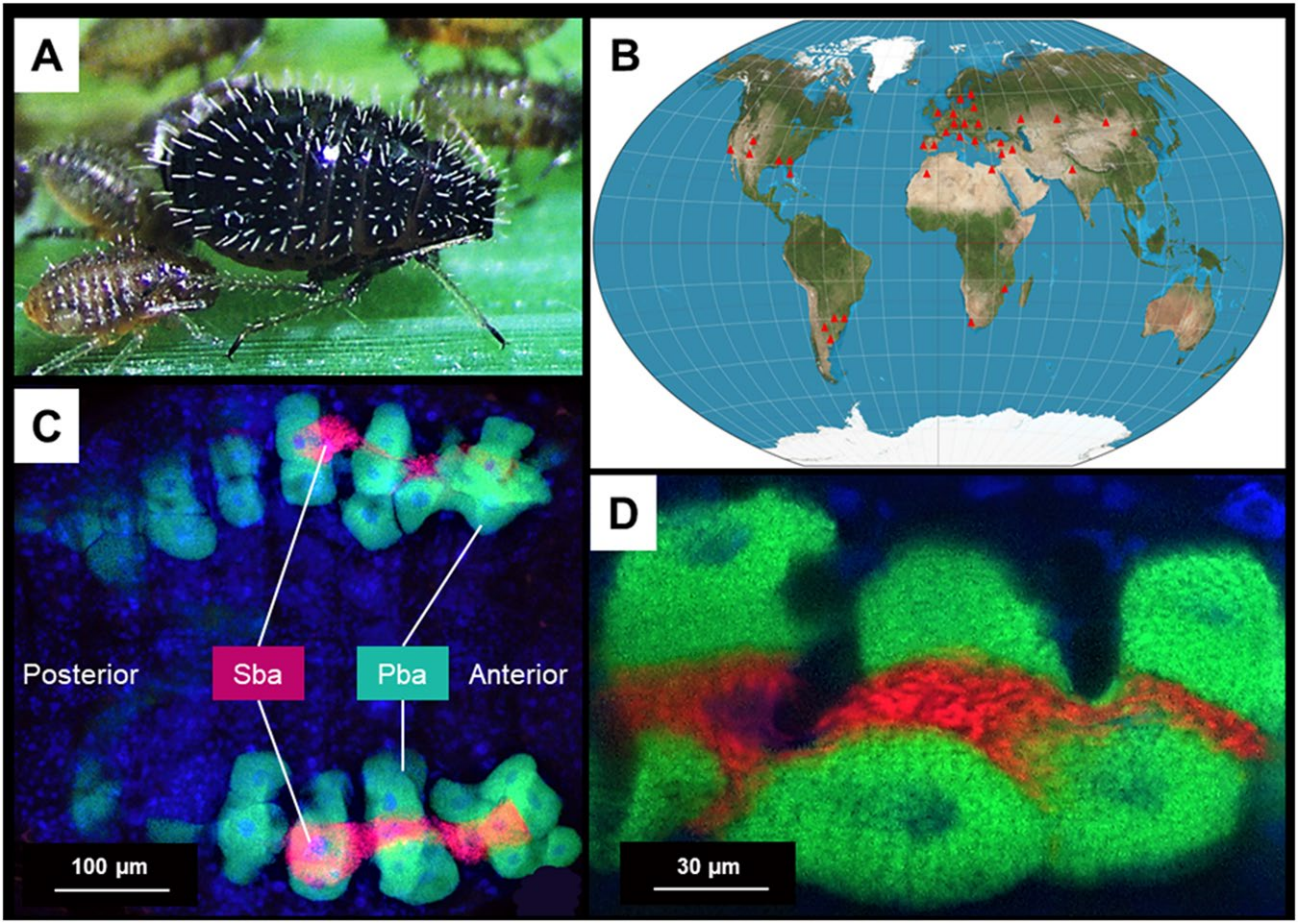
The cereal aphid *Sipha maydis* and its di-symbiotic system. (**A**) An adult surrounded by several nymphs, all feeding on bread wheat, *Triticum aestivum*. (**B**) Distribution of *S. maydis* worldwide. Red triangles represent collection locations reported in the literature. (**C**) *Serratia symbiotica* (red) is compartmentalized into syncytial secondary bacteriocytes (Sba) sandwiched between the uninucleate primary bacteriocytes (Pba) housing *Buchnera aphidicola* (green), forming a horseshoe-shaped bacteriome (green, red, and blue signals indicate *B. aphidicola* cells, *S. symbiotica* cells and host insect nuclei, respectively). (**D**) Close-up view of primary and secondary bacteriocytes showing their embedded layout.

These multi-partner symbiotic system have evolved in a wide range of hemipteran taxa, including several insect pests, including Psylloidae (psyllids^13,17–19^), Aleyrodidae (whiteflies^20–23^), Pseudococcinae (mealybugs^8,24–26^), Auchenorrhyncha (cicadas, leafhoppers, planthoppers and treehoppers^12,27–29^), Adelgidae (adelgids^15,30–35^) and Aphidoidea (aphids^14,36–41^). However, little is known about the development and functioning of these systems. This is largely due to the fact that their study is hampered by a range of constraints such as the difficulty of rearing some of these insects (e.g. cicadas), the absence of a clonal phase enabling individuals of identical genotypes to be obtained (e.g. cicadas, psyllids, whiteflies, leafhoppers), and their very small size (e.g. psyllids, whiteflies). A candidate species that overcomes all these difficulties is the cereal aphid *S. maydis* (Chaitophorinae) (Fig. 1A), a species that feeds on many species of grass (Poaceae) and is distributed in Europe, much of Asia and has recently reached North and South America where it is considered an invasive species that can damage cereal crops (Fig. 1B)^42^. *S. maydis* is easy to collect in the field, to rear and to reproduce clonally, making it an ideal species for experimental studies. Another advantage of this aphid species is that the genomes of the ancient obligate symbiont *Buchnera aphidicola* and the more recently acquired co-obligate symbiont *Serratia symbiotica* have recently been sequenced and annotated^37^. The two nutritional symbionts are compartmentalized in distinct bacteriocytes: *S. symbiotica* is confined to large syncytial secondary bacteriocytes sandwiched between uninucleate primary bacteriocytes containing *B. aphidicola* (Fig. 1C-D). This case of dual endosymbiosis is particularly relevant for studying how nutritional symbionts dwelling in distinct but contiguous bacteriocytes can collaborate metabolically with each other and with the host. However, the study of this valuable symbiotic system suffers from a lack of genomic information on the insect host. Assembling and annotating the complete genome sequence of *S. maydis* would be a highly informative and fruitful resource for deciphering multiple aspects of the species’ biology, and in particular the processes governing cooperative life between insects and anatomically integrated symbionts that form a metabolic unit in a three-way mutualistic symbiosis.

We present here the first complete assembly of the cereal aphid *S. maydis* using a PacBio high fidelity (HiFi) approach. The final assembly is 409.54 Mb in length, with a scaffold N50 of 187.22 kb and 95.5% completeness, providing an excellent genomic resource for further research on *S. maydis*. Structural annotation reveals that the genome contains 29% repeat sequences, and 24,453 protein-coding genes. Functional annotation focused on metabolism and metabolic pathway reconstruction, identifying the 2,541 enzymes of *S. maydis* involved in 273 metabolic pathways. These genomic and metabolic data provide a unique tool for studying the influence of bacterial symbiosis on insect genome evolution, and for exploring in depth the biology of *S. maydis*, an in particular the mechanisms underpinning an interdependent tripartite collaborative life between an insect and its prokaryotic partners.

## Methods

### Sample collection and genome sequencing

A colony of *S. maydis* sampled on *Hordeum vulgare* in Midelt (Morocco) in April 2016 was used to generate a clonal line from a single individual. Aphids were reared on *Triticum aestivum* (bread wheat) under long-day conditions (16 h light, 8 h dark) in a room maintained at a constant temperature of 20 °C to ensure parthenogenic reproduction. Thirty adult individuals were used for DNA extraction. Only the heads were used to minimize DNA contamination by the symbionts *B. aphidicola* and *S. symbiotica* that are present only in the abdomen, and whose genomes have been sequenced previously from the same aphid clonal line^37^. Whole insects were first surface sterilized with 99% ethanol, rinsed with sterile water and then immersed in 70% ethanol where the heads were dissected with microscissors before being stored directly in a sterile plastic tube at −80 °C prior to DNA extraction. DNA extraction was performed using phenol-chloroform. Briefly, tissues were homogenized in 500 ml STE buffer (100 mM NaCl, 1 mM EDTA, 10 mM Tris-Cl, pH 8.0) with a sterile pestle, then treated with 25 µl SDS 10% and 3 µl proteinase K (20 mg/ml). After a two-hour incubation at 55 °C with frequent mixing, the sample was treated with 6 µl RNase (10 mg/ml) and incubated at 37 °C for 30 min. Genomic DNA was purified by two successive extractions with phenol:chloroform:isoamyl alcohol (25:24:1, v/v/v) followed by extraction with 1 vol of chloroform:isoamyl alcohol (24:1, v/v/v). Genomic DNA was then precipitated by 0.7 volumes of isopropanol. After washing the pellet with 70% ethanol, genomic DNA was recovered in TE buffer (1 mM EDTA, 10 mM Tris HCl pH 8). DNA concentrations and quality were assessed using NanoDrop (Thermo Fisher Scientific, Waltham, MA, USA), agarose gel electrophoresis and Qubit fluorometer (Thermo Fisher Scientific). Sequencing libraries were prepared using the Express Template Prep Kit 3.0 (Pacific Biosciences, Menlo Park, USA) and whole-genome sequencing was performed on the PacBio Sequel IIe system at the Genomics Core Leuven (KU Leuven, Leuven, Belgium) using the Sequel® II Binding Kit 3.2 (Pacific Biosciences).

### Genome assembly and evaluation

**Fig. 2:**
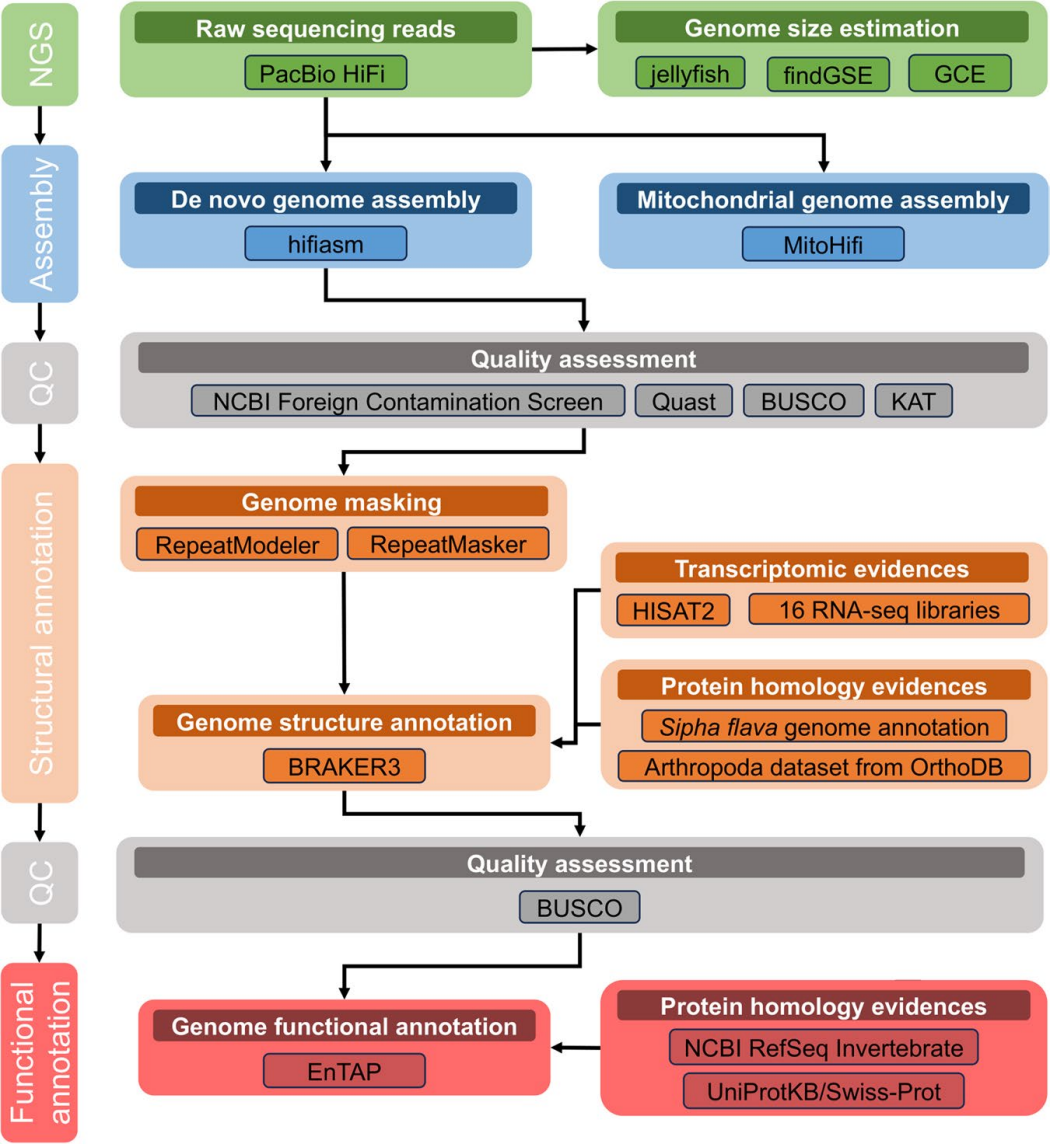
Flowchart highlighting the *Sipha maydis* genome assembly and annotation process, including quality assessment steps.

The complete *S. maydis* genome assembly and annotation workflow, including quality assessment steps, is shown in Fig. 2.

The genome size of *S. maydis* was estimated using k-mer analyses from raw PacBio HiFi reads. A k-mer (k = 21) distribution was generated with Jellyfish^43^ (v2.2.10) using the PacBio HiFi reads and genome size was estimated using three different strategies: i) findGSE^44^ v1.94.R, ii) gce^45^ v1.0.2 and iii) the ratio of total distinct k-mers divided by the frequency mode of the k-mer distribution using R^46^ v4.2.1 as described in Hon *et al*.^47^. The *S. maydis* genome was assembled from the PacBio HiFi raw reads using hifiasm^48^ v0.18.9-r527 with default parameters. The primary assembly was then screened for contaminants using the NCBI Foreign Contamination Screen (FCS) and seven contaminant scaffolds corresponding to the two endosymbiont genomes (*B. aphidicola* and *S. symbiotica*) were removed from the assembly prior to the annotation step. The accuracy and completeness of the assembly were assessed using (i) QUAST^49^ v5.0.2 with the –large and -k options, (ii) BUSCO v5.4.6^50^ using the Insecta ODB10 database, and (iii) KAT^51^ v2.4.2 to compute shared k-mers between PacBio HiFi reads and the assembly. A total of 3.70 Gb of PacBio HiFi reads with a mean read length of 6.89 kb were assembled to generate a 409.54 Mb draft genome assembly consisting of 3,570 contigs with a N50 length of 187.22 kb and a largest contig of 1.25 Mb (Table 1).

**Table 1:** Genome assembly and annotation statistics of *Sipha maydis* as compared to its close relative *Sipha flava*.

Metrics	*Sipha maydis* (this study)	*Sipha flava* (GCF_003268045.1)
**Total length (Mb)**	409.54	353.18
**No. of scaffolds/contigs**	3,570	1,923
**Scaffold/Contig N50 (kb)**	187.22	1,686.65
**Scaffold/Contig L50**	668	67
**GC%**	29.71	30.00
**No. of protein-coding genes**	24,466*	13,575
**Mean gene length (kb)**	4.98	13.00

^*^ Including the 13 mitochondrial protein-coding genes.

The assembly size is comparable to the genome size estimate of ~433 Mb using k-mers (findGSE: 446.20 Mb; gce: 421.93 Mb; total distinct k-mers divided by the frequency mode of the k-mer distribution: 431.32 Mb). The genome assembly was found to have a high level of completeness (95.5%). Of the 1,367 Insecta BUSCOs, 91.0% were complete and single-copy, 4.5% complete and duplicated, 0.8% fragmented and 3.7% were missing (Table 2). The alternative haplotype-resolved assemblies produced by Hifiasm have however a reduced total length (347.06 Mb and 329.15 Mb), N50 (70.77 kb and 71.73 kb) and complete BUSCO scores (82.6% and 81.3%) (available at: 10.57745/6RYSBE^52^).

**Table 2:** BUSCO assessment of *Sipha maydis* genome assembly compared with its close relative *Sipha flava* (Insecta_odb10 scores, n:1,367).

Metrics	*Sipha maydis* (this study)		*Sipha flava* (GCF_003268045.1)	
	Assembly	Predicted gene models	Assembly	NCBI *Sipha flava* Annotation Release 100
**Complete BUSCOs (%)**	95.5	94.8	94.3	94.7
**Complete Single-Copy BUSCOs (%)**	91.0	90.0	92.5	92.3
**Complete Duplicated BUSCOs (%)**	4.5	4.8	1.8	2.4
**Fragmented BUSCOs (%)**	0.8	1.8	0.4	0.7
**Missing BUSCOs (%)**	3.7	3.4	5.3	4.6

The mitochondrial genome was assembled using the MitoHiFi pipeline^53^ to generate a 16,379 bp genome consisting of 37 genes, including 13 protein-coding genes, 2 rRNAs, and 22 tRNAs, with a GC content of 15.43%.

### Gene prediction and general functional annotation

A *de novo* repeat library was generated using RepeatModeler^54^ v1.0.11. RepeatMasker^55^ v4.1.2 was then used with the *de novo* filtered repeat library to identify and soft-mask repeats in the draft assembly prior to annotation. Ultimately, we identified 117.21 Mb of repetitive sequences, accounting for 28.62% of the assembled genome (Table 3).

**Table 3:** Repetitive sequences in the genome of *Sipha maydis*.

Type		Numbers	Length (bp)	% of the genome
**Retroelements**	SINEs	140	35489	0.01
	Penelope	1028	298541	0.07
	LINEs	26058	13202799	3.22
	LTR elements	4983	3103658	0.76
	Total	31181	16341946	3.99
**DNA transposons**		49671	14819687	3.62
**Unclassified**		201353	68775905	16.79
**Satellites**		338	188152	0.05
**Simple repeats**		290115	12778827	3.12
**Low complexity**		45565	2225158	0.54

After masking the repeat sequences, the structural annotation (i.e., gene prediction) was performed using the BRAKER3^56–68^ pipeline v3.0.3 using *ab initio* prediction, homology searching and transcriptome-based approaches to predict protein-coding genes. For transcriptome-based prediction, the pipeline used 16 RNAseq libraries (PRJNA1031833)^69^ that were aligned to the soft-masked genome using HISAT2^70^ v2.2.1. For the homology-based approaches, annotated proteins from *Sipha flava* genome annotation (GCA_003268045.1) and the Arthropoda protein dataset from OrthoDB^71^ v11 were downloaded. The final set of protein-coding genes was retrieved from the Augustus predictions^56,57^. The completeness of the annotated protein set was assessed using BUSCO^50^ v5.4.6 and the Insecta ODB10 database. A first general run of functional annotations of predicted proteins was carried out using EnTAP^72^ v0.10.8-beta. Comparisons were performed against UniProtKB/Swiss-Prot^73^ and NCBI RefSeq invertebrate annotated proteins (https://ftp.ncbi.nlm.nih.gov/refseq/release/invertebrate/) as reference databases. Hence, we identified and soft-masked 28.62% (117.21 Mb) of the *S. maydis* genome as repeated sequences. After masking those repeated sequences, a total of 24,453 protein-coding genes were predicted using a combination of *ab initio*, homology-based and transcriptome-based approaches. The completeness of the gene prediction revealed that 94.8% of BUSCO genes were successfully detected (90.0% are single-copied and 4.8% are duplicated).

### Functional annotation of metabolism

We used several methods to perform a functional annotation of the *S. maydis* enzyme set: (i) the online KAAS – KEGG^74^ v2.1 automatic annotation server against both “For gene” and “For eukaryotes” representative sets, (ii) the v2 of the PRIAM^75^ tool, (iii) the Blast2GO^76^ pipeline v3.5 and (iv) the InterProScan^77^ v5.56 pipeline with a local installation for faster data generation. These methods generated information such as EC numbers, KEGG Orthology and Gene Ontology related to the protein sequences. All annotations were collected in a SQL database using CycADS^78^ and associated with the genomic information data. Default settings were used for software configurations and the BLAST alignments (prior to the Blast2GO analysis) were performed against the curated UniProtKB/Swiss-Prot^79^ protein sequence database.

### Metabolic network reconstruction

The final step in assessing the quality of the *S. maydis* genome was the reconstruction of its metabolic network, which validated the functional annotation of this organism’s enzyme set. This expert reconstruction step also makes the network directly accessible to the scientific community through the exploration of the dedicated metabolic database we make publicly available, or as an additional dataset that can be uploaded by users in suitable formats (e.g., sbml and Biopax). The enriched gene records containing all annotations were extracted from the CycADS SQL to generate the corresponding BioCyc-like metabolic database using Pathway Tools v27.0^80,81^, which we named SipmaCyc, according to current convention. In the summary section for each gene/protein resulting page, the information relative to the annotation results was recorded to allow the researchers to evaluate the confidence for each putative function assigned to a protein. Incomplete EC_numbers (i.e., classes and subclasses) and EC numbers inferred from a single Blast2GO annotation were excluded from the network, even though the annotation remains accessible for users in the gene description.

**Fig. 3:**
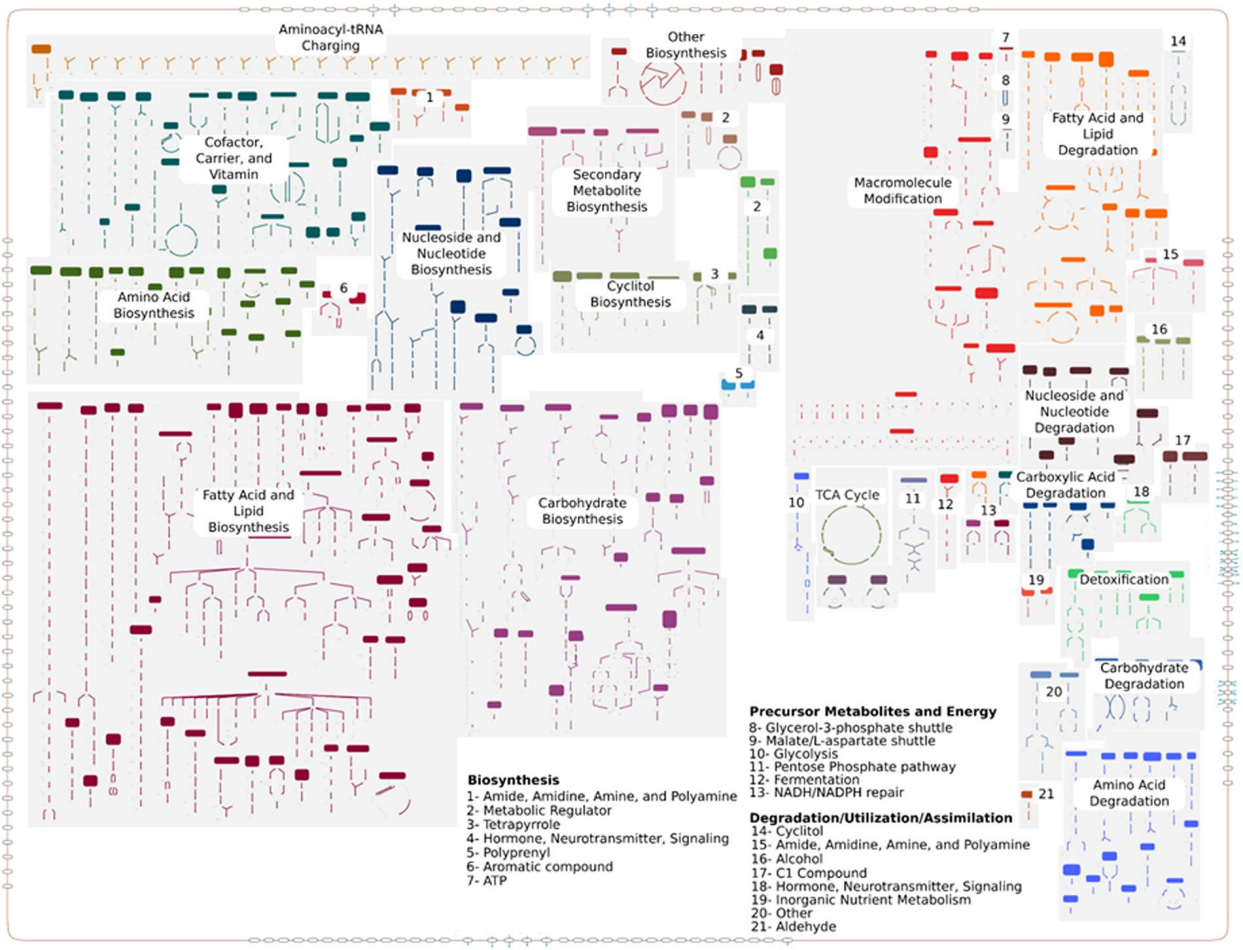
Schematic overview of the *S. maydis* metabolic network. The figure contains all the 38 metabolic categories, of which only 21 are highlighted for a more reader-friendly representation. Users can explore this map in ArtSymbioCyc, also having access to all reactions and metabolites in each pathway.

Since the functional annotation of metabolic pathways in an insect dependent on nutritional symbionts cannot be done without taking into account their metabolic contributions, we validated the *S. maydis* metabolic annotations by assessing their homogeneity and correct integration with those of its symbiotic partners, *B. aphidicola* and *S. symbiotica*, using the CycADS annotation system^78,82^. This led to the production of an integrated metabolic network of *S. maydis* and its bacterial associates, which we made publicly available on ArtSymbioCyc^82^ (http://artsymbiocyc.cycadsys.org/), a collection of metabolic databases dedicated to arthropod symbioses. *S. maydis* encodes 26,059 predicted proteins from its 24,466 protein-coding genes, of which 2,523 are enzymes involved in 273 metabolic pathways. The SipmaCyc database in ArtSymbioCyc provides a complete description of the central metabolism of *S. maydis* at the genome scale (Fig. 3**)** and enables users to visualize and explore individual metabolic networks at the level of compounds, reactions, or pathways.

## Data Records

The sequencing data that were used for the genome assembly and annotation have been deposited in the NCBI Sequence Read Archive with accession numbers SRP443918^83^ and SRP468263^69^ respectively. This genome assembly is available under accession number GCA_034509805.1^84^.

Data on metabolic network reconstructions are available at Recherche Data Gouv (10.57745/6RYSBE)^52^ as well as in the ArtSymbioCyc collection (http://artsymbiocyc.cycadsys.org/)^82^. Metabolic network reconstructions and the resulting BioCyc metabolism databases are available in the ArthropodaCyc collection^82^ (https://arthropodacyc.cycadsys.org) for *S. maydis* alone (organism database: “*Sipha maydis*”) and in the ArtSymbioCyc collection (http://artsymbiocyc.cycadsys.org/) for *S. maydis* associated with its two obligate nutritional symbionts (organism databases: “*Sma-Sipha maydis*” and “*Sma-Sipha maydis holobiont*”). A single repository (Recherche Data Gouv)^52^ was created to unite (i) the *S. maydis* genome assembly commands file (txt), (ii) the genomic files (fasta primary and haplotype-resolved assemblies), (iii) the gtf/gb structural annotations for both the genome and the mitochondrion and (iv) the functional annotations (tabular text), reactions (sbml) and network in Biopax format for the three partners composing the symbiotic system.

## Technical Validation

The quality of the *S. maydis* genome assembly was assessed by computing several metrics: (i) comparison with the estimated genome size, which is ~433 Mb (see above); (ii) genomic BUSCO analyses, which identified 95.5% of Insecta BUSCOs in the *S. maydis* genome (91.0% are single-copied and 4.5% are duplicated, Table 2), and 94.8% of Insecta BUSCOs proteins in its predicted gene models (90.0% are single-copied and 4.8% are duplicated, Table 2); (iii) comparison with the PacBio HiFi reads using QUAST and KAT which showed that 99.95% of the k-mers (k = 27) of our assembly are covered by the k-mers from the PacBio HiFi reads and 99.73% of the PacBio HiFi reads could be mapped into the assembly. Despite a low sequencing yield (3.70 Gb) and small read lengths (6.89 kb), the aforementioned quality metrics indicated that the *S. maydis* genome assembly has a high level of completeness and is of high-quality.

The functional annotation of the *S. maydis* genome coding for central metabolism is supported by the use of our CycADS expert system and leads to the possibility of reconstructing the metabolic network, whose integrity and consistency can be tested using the comparative tools of the ArtSymbioCyc database interface^82^. As an example, Table 4 shows the distribution of *S. maydis* reactions in the top 6 levels of the Enzyme Commission classification, which is fully consistent with those of the other insects in the database.

**Table 4:** Distribution of *S. maydis* reactions across the 6 top-level categories identified by the Enzyme Commission.

EC Category*	*Sipha maydis* (this study)
**1–Oxidoreductases**	439 (23%)
**2–Transferases**	709 (38%)
**3–Hydrolases**	435 (23%)
**4–Lyases**	133 (7%)
**5–Isomerases**	69 (4%)
**6–Ligases**	97 (5%)
**7–Translocases**	44 (2%)
**Total reactions with full or partial EC Numbers**	1,882

^*^Included in this table are all reactions in the database which have been assigned either full or partial EC numbers, and for which an enzyme has been identified.

## Data Availability

All software and pipelines were executed according to the manual and protocols of the published bioinformatic tools. The version and code/parameters of software have been described in the Methods section. Metabolic network reconstructions were carried out using pathway tools 27.0 (April 12, 2023), with annual updates planned.
